# Cold Plasma as an Innovative Construction Method of Voltammetric Biosensor Based on Laccase

**DOI:** 10.3390/s18124086

**Published:** 2018-11-22

**Authors:** Szymon Malinowski, Cecylia Wardak, Justyna Jaroszyńska-Wolińska, P. Anthony F. Herbert, Rafał Panek

**Affiliations:** 1 Faculty of Civil Engineering and Architecture, Lublin University of Technology, Nadbystrzycka 40, 20-618 Lublin, Poland; j.wolinska@gmail.com (J.J.-W.); r.panek@pollub.pl (R.P.); 2 Department of Analytical Chemistry and Instrumental Analysis, Faculty of Chemistry, Maria Curie-Sklodowska University, Maria Curie-Sklodowska Sq. 3, 20-031 Lublin, Poland; cecylia.wardak@poczta.umcs.lublin.pl; 3 Plasma Ireland, 22 Summerhill North, T23 N592 Cork, Ireland; therbert@irishprecisionoptics.com

**Keywords:** laccase biosensor, plasma polymerization, corona discharge, bio-recognition layer coating, rutin determination

## Abstract

Development of new, faster methods of biosensor construction is a huge challenge for current science and industry. In this work, biosensor construction was carried out using a new soft plasma polymerization (SPP) method in which a bio-recognition layer of laccase enzyme was polymerized and bonded to a glassy carbon electrode (GCE) substrate under atmospheric pressure with a corona discharge jet. Laccase belongs to the oxidoreductase enzyme group with four copper atoms in its active center. Application of the corona SPP plasma method allows reduction of the time needed for biosensor construction from several hours to minutes. The presented work includes optimization of the laccase bio-recognition layer deposition time, structural studies of the deposited laccase layer, as well as study of the fabricated biosensor applicability for the determination of Rutin in real pharmaceutical samples. This method produces a biosensor with two linear ranges from 0.3 μmol/dm^3^ to 0.5 μmol/dm^3^ and from 0.8 μmol/dm^3^ to 16 μmol/dm^3^ of Rutin concentration. Results shown in this work indicate that application of the one-step, corona SPP method enables biosensor construction with comparable analytical parameters to biosensors fabricated by conventional, multi-step, wet methods.

## 1. Introduction

Biosensors are increasingly popular in the quantitative determination of many chemical compounds and heavy metals. They are one of the class of chemical sensors with a bioactive recognition (bio-recognition) layer composed of enzymes, antibodies, DNA or RNA [1]. Among them, historically first and the most popular, are biosensors composed of enzyme-recognition layers [2] where the analytical signal is received as a result of enzymatic reaction with the determined chemical compound [3]. Their popularity comes from the simple procedures of enzyme modification and its good catalytic properties [2]. Enzyme-based biosensors are characterized by excellent selectivity but shorter life-time and inconsistent activity of the bio-recognition layer [3].

In the construction of biosensors by the soft plasma polymerization (SPP) method presented in this paper, Laccase enzyme (polyphenoloxidase; EC 1.10.3.2) was used in the form of biological precursor. It belongs to the blue multi-copper-oxidase family and is generated by plants, wood-rotting fungi as well as saprophytic ascomytes [4]. The active center of laccase is composed of three main parts containing copper atoms: type I, type II and type III. Because of their properties, biosensors based on laccase recognition layers are very often chosen for quantitative analysis in the food, textile and paper industries, environmental protection as well as in medical analysis. In comparison to other kinds of bio-recognition layers, the application of laccase allows electron transfer without additional co-factors in the quantitative determination of many phenolic compounds as a result of their oxidation by molecular oxygen [2].

Rutin (rutoside, quercetin-3-rutoside) with the chemical formula 3,3′,4′,5,7 -pentahydroxyflavone-3-rhamnoglucoside belongs to the flavonoid group [5,6], one of the main dietary constituents of humans [7]. Rutin is a polyphenolic compound [8] with several hydroxyl groups attached to a C3–C6–C3 ring and is produced by numerous plants [8] such as passion flower, buckwheat, tea and apple [5]. The flavonoids group is composed of more than 4000 chemical compounds categorized into flavonols, flavones, flavanones, catechins, anthocyanidins and chalcones classes [7]. The name “Rutin” originates from the plant *Ruta graveolenes*, which is a common source of this flavonoid. From the chemical point of view, Rutin is a glycoside composed of flavonoic aglucone quercetin along with disaccharide rutinose [5]. This compound is used in pharmacy and medicine as anti-inflammatory, antibacterial, anti-ageing and antioxidant therapeutic agents [9] because it decreases the amount of various oxidizing species such as superoxide anions, hydroxyl radicals or peroxyl radicals [10].

Quantitative determinations of Rutin currently employ many instrumental methods such as chemiluminescence, high-performance liquid chromatography, spectrophotometry as well as electrochemistry. Most of these techniques need complicated pre-concentration procedures and instruments and, thus, are time-consuming and expensive [6]. Preferable are electrochemical methods [11] characterized by high sensitivity, good stability, simplicity and low instrument costs [12]. Increasingly in Rutin determination biosensors, an electrochemical method is used. Their application allows faster and more versatile quantitative analysis [13]. In the electrochemical determination of Rutin many different sensors and biosensors have been developed such as: hanging mercury drop electrode (HMDE) [14,15], modified glassy carbon electrode (GCE) [16], modified carbon paste electrode [10], modified gold electrode [17,18,19] and modified carbon ceramic electrode. Currently known electrode modification pathways are characterized by complex and time-consuming procedures involving many organic, often toxic solvents and modifiers. In the modification of electrodes different nanomaterials have been used such as noble metal nanomaterials, metal oxide nanomaterials, carbon nanomaterials, polymers and bio-nanomaterials [20]. One of the nanomaterials used in electrode modification is gold nanoparticles (AuNPs). These have the good conductivity needed for sensor/biosensor construction. AuNPs have been applied in work [12] in an electrode modification procedure comprising three main steps: modification of the GCE by multi-walled carbon nanotubes, electrografting of ethylenediamine, and electrostatic assembly of AuNPs. Other nanoparticles such as CeO_2_ have been used for electrode modification by [21]. Apart from nanomaterials in Rutin sensor construction, ionic liquids (ILs) are used. These are molten salts consisting of ions which melt at temperatures <100 °C and act as a binding agent [8] in the modification of electrodes, especially a carbon paste electrode (CPE). They are very popular in the construction of sensors/biosensors because of their high chemical and thermal stability, good conductivity and ability to dissolve polar and non-polar chemical compounds [22].

This work describes the analytical performance parameters of laccase biosensors constructed using the one-step, non-waste SPP method described in work [23,24,25]. The measured analytical performance parameters of these devices indicate that the new plasma method can simplify biosensor production enabling faster availability and lower production costs. The studies were performed on the laccase enzyme because of its wide application in bio-sensing of many organic and polyphenolic compounds as well as heavy metals. The SPP method is based on the polymerization/crosslinking of the biological precursor inside pin-to-plane corona discharge plasma with a low energy density to minimize molecular damage to the monomer. The unique properties of this plasma type, low energy, room temperature, atmospheric pressure operation, and the resulting SPP process enables simple, single-step bio-recognition layer deposition for generation of the analytical signal. The principle of the corona SPP method is polymerization/cross-linking and binding of laccase onto solid support in a corona discharge plasma reaction zone as was confirmed in [24]. Until recently, plasma polymerization was used only for deposition of highly polymerized non-bioactive coatings including functional groups, i.e., amine or carboxyl, important to biosensor construction. Application of plasma polymerization in the direct fabrication of biosensor recognition layers allows great process simplification and reduction in sensor fabrication time from several hours to a few minutes. The properties of corona discharge applied in the SPP method allow the introduction of biological precursor, here in the form of laccase enzyme, directly into plasma reaction zone without excessive molecular damage which constitutes an innovation in biosensor construction.

This paper follows work presented by the authors in references [23,24,25]. Reference [25] in 2009 introduced the SPP technique using corona jet plasma but was limited only to deposition of high molecular weight organic compounds with no biological component. In reference [24] we presented a study of corona SPP-deposited laccase bio-recognition layers characterized by molecular weight and structure (circular dichroism spectroscopy) as functions of the key SPP process control factors, namely plasma generation applied voltage, plasma power and specific energy density, helium gas flow, deposition time and laccase solution flow rate. The bio-activity response to these factors was not investigated. Finally, in reference [23] we presented the bio-active analytical performance parameters of a single laccase bio-sensor fabricated with a plasma deposition time of 15 s. This demonstrated bio-sensor functionality in the detection of Rutin but poor stability rendering it unusable in the analysis of real pharmaceutical samples. The purpose of this work, therefore, is to extend our investigation by exploring the effects of the critical deposition process control parameter, plasma deposition time, on the analytical performance parameters of corona SPP-fabricated laccase bio-sensors with the aim of bringing them closer to viability in the testing of real Rutin samples. Indeed, in this paper we demonstrate that increase of deposition time significantly affects the analytical parameters of the biosensors enabling greater suitability for analytical Rutin determination in real pharmaceutical samples. This paper also looks at the effects on sensor performance of the supporting electrolytes, the pH of the buffer solutions, and storage conditions as well as any interference effects due to co-existing species in the Rutin solution.

## 2. Materials and Methods

### 2.1. Chemicals

All chemicals were analytical grade or Suprapur and used without further purification. Ultrapure water obtained from Milli-Q purification system was used for solutions preparation. A Suprapur acetic acid (CH_3_COOH) and sodium hydroxide (NaOH) obtained from Merck were used for preparation of acetate buffer. Rutin, sodium hydrogen phosphate (Na_2_HPO_4_), sodium dihydrogen phosphate (NaH_2_PO_4_), and potassium phthalate (KHC_8_H_4_O_4_) were purchased from Sigma Aldrich. Rutin stock solution at concentration 1 mmol/dm^3^ was prepared by dissolution of the appropriate amount of Rutin in ethanol and immersed in an ultrasonic bath to obtain clear and homogenous solutions. The working solutions of Rutin of lower concentrations were prepared by dilution of the stock solution as required.

Laccase from Cerrena unicolor C-139 enzyme was obtained from the culture collection of the Ragensburg University and deposited in a fungal collection of the Department of Biochemistry, Maria Curie-Sklodowska University. Laccase isolation was carried out using procedures described in [26]. Working solutions of laccase have been prepared by 10-fold dilution of the laccase solution prepared by the dissolution of laccase liophylizate in 1 mL of deionized water. The initial concentration of laccase liophylizate was C_Laccase_ = 178 mg/mL and activity was 186,000 nkat/L. After lyophilizing, the laccase activity dissolved in 1 mL of MilliQ water was 2,350,100 nkat/L (141 U/mL) per/mg of protein.

### 2.2. Preparation of Rutin Pharmaceutical Sample

Fabricated biosensors were applied in the determination of Rutin pharmaceutical samples. Samples of pharmaceuticals containing Rutin (Rutinoscorbin, Cerutin, Vanescin) were prepared by dissolution of tablets in 5 mL of 96% ethyl alcohol solution in an ultrasonic bath and made up to 10 mL with acetate buffer at pH = 5.00. The obtained solutions of pharmaceutical Rutin were filtered directly before measurement.

### 2.3. Biosensor Construction

Glassy carbon electrodes were polished using 0.3 μm alumina slurry. Then, the electrodes were ultra-sounded several times in water and methanol and covered by the active enzyme layer using the one-step corona SPP method as described in [24]. During plasma deposition of the recognition layer, liquid solution laccase precursor was injected at a constant flow rate of 200 μL/min using syringe pump (SyringePump, New Era Pump Systems, Inc., New York, NY, USA) into a nebulizer (Burgener type T2100, Mississauga, ON, Canada) in order to transform the liquid into a vapor or quasi-vapor form. The atomized laccase vapor was injected into a helium gas stream and carried into the plasma zone of a low-energy pin-to-plane corona discharge. Due to the actions of the active, energetic plasma species (electrons, ions, excited states) the laccase monomer was polymerized and/or crosslinked into the bio-recognition layer deposited on the GCE surface. This paper presents the analytical performance parameters of three different laccase biosensors named as: GCE/laccase_10_, GCE/laccase_15_, GCE/laccase_30_ deposited respectively for 10, 15, and 30 s by the corona SPP process in optimized plasma conditions [24] at applied voltage of 3 kV and helium flow rate of 10 L/min at room temperature and at atmospheric pressure.

### 2.4. Electrochemical Measurements

Electrochemical measurements were performed by Electrochemical Analyzer EA-9 (MTM-ANKO, Cracow, Poland) using the square-wave voltammetric method. The electrochemical cell was assembled using the conventional three-electrode system where the new laccase-based biosensor was used as the working electrode, Ag|AgCl (3 mol/dm^3^ KCl) electrode as the reference electrode and a platinum electrode as an auxiliary electrode. Square-wave voltammograms were recorded from 0 to 600 mV; other parameters were as follows: pulse amplitude 20 mV, frequency 40 Hz, scan increment 5 mV. All electrochemical experiments were carried out at room temperature (25.0 ± 0.5 °C) in non-de-aerated, unstirred solution using an electrochemical cell volume of 10.00 mL.

### 2.5. Scanning Electron Microscope (SEM) Images

The morphological structures of laccase bio-recognition layers deposited for 10, 15 and 30 s by the SPP process were determined means of a scanning electron microscope (SEM) FEI Quanta 250 FEG (FEI, Hilsboro, OR, USA) equipped with a system of chemical composition analysis based on energy dispersive spectrometry (EDS) X-ray-EDS from the EDAX company (EDAX Inc., Mahwah, NJ, USA).

## 3. Results

Unmodified GCE with no laccase bio-coating was first investigated for its analytical response to Rutin. This was done in a blind test and in three increasing Rutin concentrations 1 μmol/dm^3^, 5 μmol/dm^3^ and 10 μmol/dm^3^. It was found that an unmodified GCE electrode does not respond to Rutin. Both in the blind test and the varying Rutin concentrations the same voltammograms were obtained. Rutin does not demonstrate redox activity with unmodified GCE, so modification of a GCE electrode surface, e.g., using a laccase bio-recognition layer, is necessary.

### 3.1. SEM Images of Soft Plasma Polymerization (SPP) Deposited Laccase Biosensors

**Figure 1:**
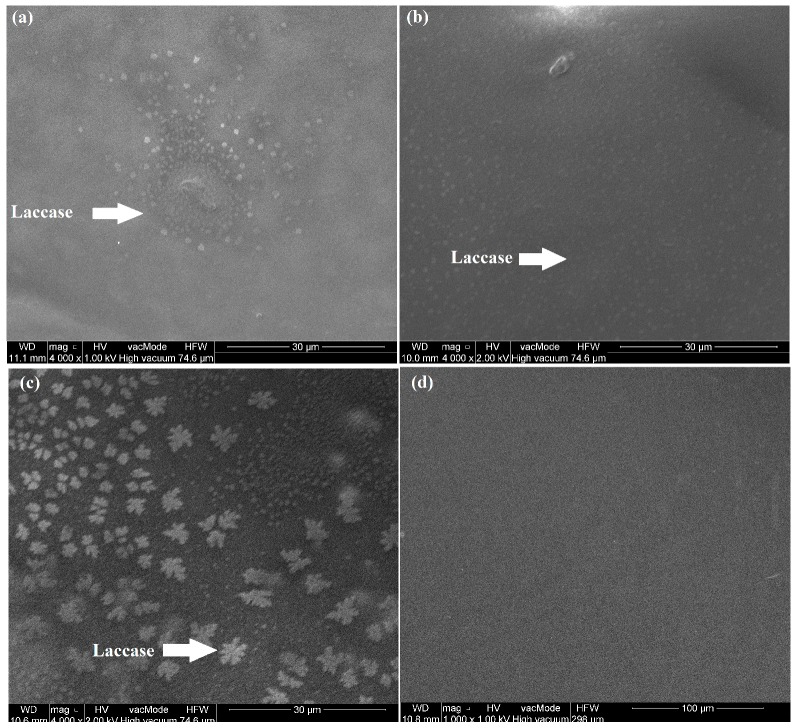
Scanning electron microscope (SEM) images of laccase layers deposited for (**a**) 10 s, (**b**) 15 s, (**c**) 30 s by corona soft plasma polymerization (SPP) deposition process and (**d**) unmodified glassy carbon electrode (GCE).

As is seen in Figure 1, the laccase bio-recognition layer deposited inside the corona discharge jet is non-uniform and consists of laccase clusters distributed over the GCE electrode surface. SEM imaging of the laccase bio-recognition layers deposited by 10, 15 and 30 s plasma processing showed that the laccase clusters size and coverage are strongly dependent on the time of SPP deposition. Figure 1a shows that application of the 10 s SPP process generates the laccase bio-recognition layer in the form of several large clusters of laccase molecules. Extension of the SPP process time to 15 and 30 s results in deposition of the bio-recognition layer in the form of larger clusters uniformly distributed over the whole of the GCE electrode surface. Moreover, 30 s deposition of laccase results in clusters with a more complex structure.

### 3.2. Optimization of SPP Deposition Time

**Figure 2:**
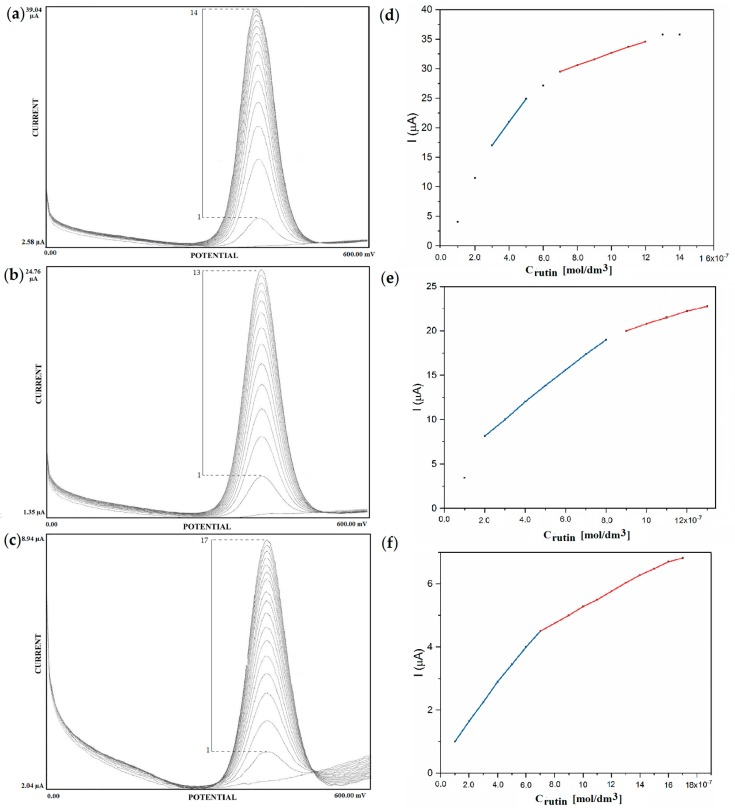
Square-wave voltammograms for Rutin solutions at the following concentrations: (1) 0.1, (2) 0.2, (3) 0.3, (4) 0.4, (5) 0.5, (6) 0.6, (7) 0.7, (8) 0.8, (9) 0.9, (10) 1.0, (11) 1.2, (12) 1.3, (13) 1.4, (14) 1.5, (15) 1.6, (16) 1.7, (17) 1.8 (µmol/dm^3^) (**a**) of GCE/laccase_10_, (**b**) GCE/laccase_15_, (**c**) GCE/laccase_30_ and calibration curves of (**d**) GCE/laccase_10_, (**e**) GCE/laccase_15_, (**f**) GCE/laccase_30._

The effect of SPP deposition time was studied by determination of biosensor response based on anodic peak current in Rutin solutions of the concentration range 0.1–2.0 μmol/dm^3^. Optimization of deposition time has been evaluated on the basis of the linear range of responses, sensitivity and signal stability for three different biosensors, i.e., GCE/laccase_10_, GCE/laccase_15_ and GCE/laccase_30_ constructed according to the procedure described in Section 2.3. The linearity of biosensors was recorded in an environment of acetate buffer at pH = 5.00 for increasing Rutin concentration in steps of 0.1 µmol/dm^3^. Figure 2a–c shows square-wave voltammograms and corresponding calibration graphs obtained for GCE/laccase_10_, GCE/laccase_15_, and GCE/laccase_30_, respectively. All of the studied biosensors show two linear ranges at different Rutin concentrations.

As is seen in Figure 2a, GCE/laccase_10_ has a linear range from 0.3 μmol/dm^3^ to 0.5 μmol/dm^3^ and from 0.7 μmol/dm^3^ to 1.2 μmol/dm^3^ with linear equation and correlation coefficients Equations (1) and (2) respectively.

ΔI = 39.25C_Rutin_ + 5.25  R^2^ = 0.999 (1)

ΔI = 10.32C_Rutin_ + 22.32  R^2^ = 0.999 (2)

As is seen in Figure 2b, GCE/laccase_15_ has a linear range from 0.2 μmol/dm^3^ to 0.8 μmol/dm^3^ and from 0.9 μmol/dm^3^ to 1.3 μmol/dm^3^, with linear equation and correlation coefficients Equations (3) and (4) respectively.

ΔI = 18.18C_Rutin_ + 4.63  R^2^ = 0.998 (3)

ΔI = 7.42C_Rutin_ + 13.35  R^2^ = 0.996 (4)

As is seen in Figure 2c, GCE/laccase_30_ has a linear range from 0.1 μmol/dm^3^ to 0.7 μmol/dm^3^ and from 0.7 μmol/dm^3^ to 1.3 μmol/dm^3^, with linear equation and correlation coefficients Equations (5) and (6) respectively.

ΔI = 5.85C_Rutin_ + 0.48  R^2^ = 0.998 (5)

ΔI = 2.47C_Rutin_ + 2.78  R^2^ = 0.999 (6)

Linear range is directly related to the sensitivity of the biosensor obtained from the slope of the analytical curve. As seen in Figure 2a–f, the sensitivity of biosensors deposited by the corona SPP method is strongly affected by deposition time of the laccase bio-recognition layer. In addition, different sensitivities of the biosensors were obtained for their first and second linear ranges. Figure 2 shows that the highest sensitivity values are respectively 34.43 (µA·dm^3^/µmol) and 7.36 (µA·dm^3^/µmol) for the 1st and 2nd linear ranges of GCE/laccase_10_. Extension of laccase bio-recognition deposition time to 15 s caused decreasing biosensor sensitivity to 17.96 (µA·dm^3^/µmol) and 6.99 (µA·dm^3^/µmol) for the 1st and 2nd linear ranges, respectively. Tripling deposition time resulted in biosensor sensitivity almost 6 times lower in the 1st linear range and approximately 3 times lower in the 2nd linear range.

**Figure 3:**
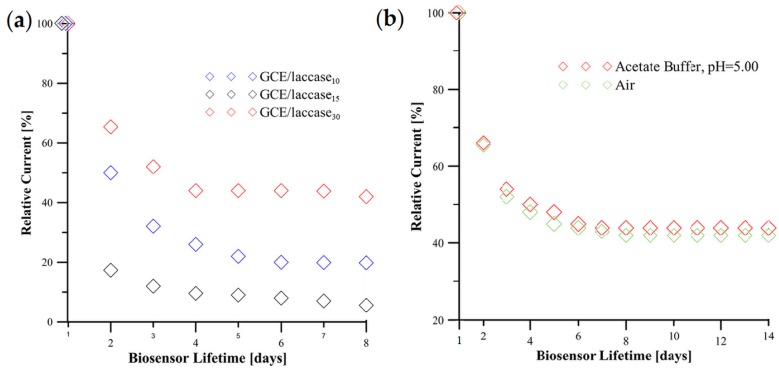
(**a**) Relative current (%) of GCE/laccase_10_, GCE/laccase_15_ and GCE/laccase_30_ vs. its lifetime (days) and (**b**) relative current (%) of GCE/laccase_30_ stored in different storage conditions.

Apart from linear range and sensitivity, an important factor in the applicability of the biosensor is the stability of the analytical signal. The stability of the fabricated biosensors was evaluated on the basis of their response recorded in 0.6 µmol/dm^3^ Rutin (pH = 5.00) solution. The results shown in Figure 3a indicate that the relative currents (%) of GCE/laccase_10_, GCE/laccase_15_ and GCE/laccase_30_ decrease over 8 days respectively to 19.90%, 5.57%, and 42.05%. Relative current (%) is defined as a ratio of measured current and maximum current. A similar effect was also observed in the literature [27]. This crucial factor plays an important role in practical applications.

Optimization of the SPP deposition process time is not straightforward as it requires assessment of three dependent responses, namely linear range, sensitivity and signal stability. The linear range response shows that GCE/laccase_15_ is superior. However, this biosensor is characterized by a large decrease in analytical signal of ~80% in two days making application of sensors based on this deposition time difficult to justify. From the analytical sensitivity perspective GCE/lacccase_10_ is superior but, again, signal stability is poor. Analysis of all of the responses indicates that the optimum deposition time is the 30 s GCE/laccase_30_, which, therefore, was selected for further experimentation. This biosensor is characterized by the most stable signal and widest linear range but lower analytical sensitivity. It is critical that the linear ranges of all studied biosensors are stable and invariable.

### 3.3. Study of Experimental Conditions of GCE/Laccase_30_

Development of these new biosensors also included investigation of various experimental parameters such as supporting electrolyte and its pH as well as response time and storage conditions.

**Figure 4:**
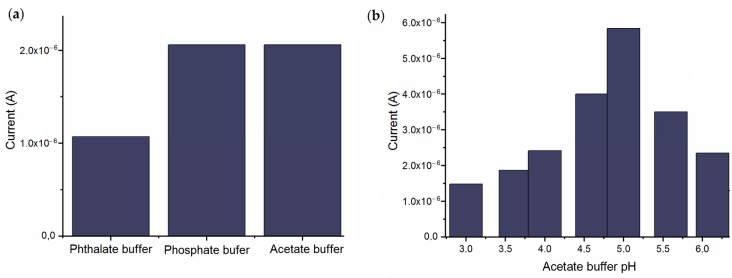
(**a**) Effect of type of buffer on current of GCE/laccase_30_ and (**b**) effect of acetate buffer pH on current of GCE/laccase_30_.

During selection of supporting electrolytes, the analytical responses of GCE/laccase_30_ have been recorded for the same Rutin concentration (C_Rutin_ = 0.4 μmol/dm^3^) in the environment of three different buffers, i.e., phthalate buffer, phosphate buffer and acetate buffer at the same pH = 5.00. Figure 4a shows that two of the studied buffers obtained 100% of current values. Thus, GCE/laccase_30_ can be successfully used in Rutin determination both in phosphate and acetate buffer environments without any loss of analytical signal in contrast to the application of phthalate buffer which causes a decrease in analytical signal of about 50%. GCE/laccase_30_ in acetate buffer was selected for further studies as this buffer is most often chosen in Rutin determination and it is more stable.

Highly significant in biosensor operation is the pH of the buffer solution. The effect of pH was investigated in an acetic acid solution pH = 3.00 and in acetate buffer solutions of pH range from pH = 3.63 to pH = 6.10 at the Rutin concentration C_Rutin_ = 0.4 μmol/dm^3^ using GCE/laccase_30_ bio-sensor. Figure 4b clearly indicates that the best analytical response of GCE/laccase_30_ was achieved for pH = 5.00 consistent with the papers [8,27,28]. Decrease in acetate buffer pH to 4.60 or increase to 5.60 resulted in the analytical signal reducing to respectively 69% and 60% of the pH = 5.00 signal.

Biosensor response time directly influences measurement time and, in consequence, their applicability in the quantitative analysis of real samples. Response times of GCE/laccase_30_ were studied at three different Rutin concentrations 0.2 μmol/dm^3^, 0.4 μmol/dm^3^ and 0.6 μmol/dm^3^ and the results showed that the response time of the GCE/laccase_30_ sensor is independent of Rutin concentration and is 120 s. Therefore, in the determination of Rutin in real, pharmaceutical samples analytical signals were recorded after this period from the moment of dosing the analyte solution.

A further factor investigated for influence on analytical response was the storage conditions of GCE/laccase_30_ at the temperature of 4 °C. Figure 3b shows that storing GCE/laccase_30_ in 1 mol/dm^3^ acetate buffer results in a smaller decrease in relative current than storing in air. Air storage results in a decrease in relative current to 42% of the original signal while storage in acetate reduces the relative current to 49% of original signal over a period of 15 days.

### 3.4. Study of the Applicability of GCE/Laccase_30_

Applicability of the GCE/laccase_30_ biosensor was evaluated by determination of Rutin in real, pharmaceutical tablet samples. The analysis was performed by the standard addition technique. The results obtained by the as-fabricated biosensor were compared with those obtained by the ultraviolet-visible (UV-Vis) spectrophotometric method and with labelled content. As is seen in Table 1, biosensor measurements of the tested tablets’ Rutin received recovery varied from 96.8% to 104.0%. There is good agreement between the Rutin content in the tested pharmaceuticals determined by two independent methods and the declared amount. Such agreement shows that the performance of the GCE/laccase_30_ biosensor appears to be largely independent of the sample.

### 3.5. Interference Studies

The influence of coexisting species on the analytical signal of GCE/laccase_30_ has been also investigated. The studies were performed at constant Rutin concentration 1 μmol/dm^3^ in the presence of 5 times larger concentrations of coexisting species such as: lactose, glucose, sodium chloride, starch, glycine, glutamic acid, mannitol and glutaric acid. The received results showed that these substances at the studied concentrations do not affect the analytical signal of GCE/laccase_30_. It clearly indicates that the corona SPP biosensor obtained is free from interference by most organic substances commonly used in pharmaceutical preparations.

## 4. Discussion

Laccase-based biosensors are popular in many industries as well as in environmental protection. Currently, their construction procedures are long and involve many organic chemical compounds in the form of cross-linkers, etc. so that there is need for the development of faster and easier methods of their construction. One such method may be SPP based on the polymerization of biologically active precursor inside a corona discharge plasma jet with low energy density. Plasma polymerization is a well-known and commonly used technique of deposition of thin, functional coatings including biologically active coatings such as laccase enzyme. Therefore, this method can potentially be applicable in biosensor construction by deposition of bio-recognition layers. The aim of this work was the investigation of the measurement and working conditions as well as the analytical performance parameters of biosensors in which laccase bio-recognition layers were deposited using the corona SPP method and their application in Rutin determination in pharmaceutical samples. Laccase bio-recognition layers are very popular for biosensor construction because of the redox properties of the laccase enzyme enabling determination of multiple chemical compounds. According to the literature [29], determination of Rutin and other redox molecules occurs via simultaneous reduction of the di-oxygen molecule to water. During the enzymatic electrode reaction Rutin is electrochemically oxidized to o-quinone, which is subsequently reduced back to Rutin at a potential of +0.35 V [8,30]. Critical in the selection of analyte for bio-electrochemical determination is stability both in its oxidized and reduced form.

Development of biosensors with lower levels of linearity allows their application in determination of chemical compounds at lower concentration levels. As is seen in Figure 2a–c, all the corona SPP deposited laccase biosensors (GCE/laccase_10_, GCE/laccase_15_, GCE/laccase_30_) have two linear ranges with good correlation coefficients (R^2^ > 0.996). Comparison of the linear ranges of laccase biosensors deposited by the corona SPP method and other biosensors designed for Rutin determination is presented in Table 2. These data clearly show that biosensors from this work are characterized by linear ranges at much lower level concentrations of Rutin with higher sensitivities than current Rutin sensors.

Results show that a corona discharge plasma jet can be successfully applied in biosensor construction as laccase bio-recognition layers deposited using the SPP method retain their original redox properties. It appears that the laccase plasma polymerization process occurs only through external bonds of laccase rather than the enzyme active center responsible for the generation of the analytical signal. Thus, corona SPP delivers cross-linking of the laccase without damage to or denaturing of the essential core of the monomer molecule. The results indicate that the corona SPP method can replace conventional longer and more costly methods of biosensor fabrication whilst retaining adequate analytical performance parameters.

On the basis of Figure 2a–c it can be said that applied deposition time strongly influences the sensitivity of SPP deposited laccase biosensors. Deposition time directly determines the amount of laccase in the as-deposited layer but also the contact time of deposited laccase molecules with the high energy active species of the corona discharge plasma jet. Extension of deposition time from 10 s to 30 s causes decreased sensitivity in both the 1st and 2nd linear ranges despite the increased amount of as-deposited laccase. This is almost certainly due to molecular damage and deactivation of previously deposited laccase through bombardment by active species such as ions, electrons, radicals and molecular fragments.

Many different factors impact the stability of the analytical signal of biosensors fabricated by the corona SPP method. The most important can be partial deactivation of the laccase bio-recognition layer during biosensor work. As mentioned, determination of Rutin occurs by reduction of laccase and oxidation of Rutin [10]. Decrease in the analytical signal of laccase biosensors deposited by SPP can be caused by the absence of or incomplete laccase re-oxidation of the bio-recognition layer. The oxidized form of laccase is not able to perform oxidation of Rutin and, thus, generate an analytical signal.

Corona SPP-fabricated biosensor stability is strongly dependent on deposition time of the laccase coating. Deposition time directly affects the quantity of laccase deposited and the degree of polymerization of the laccase precursor. Figure 3a shows that extension of deposition time resulted in smaller decrease of analytical signal from the biosensor over time. The increased quantity of active laccase in the as-deposited bio-recognition layer together with a higher degree of polymerization and crosslinking of the laccase monomer possibly conferring greater coating stability seem likely to contribute to reduced loss of analytical signal over time. The nature of the signal decrease as shown in Figure 3a would seem to give an indication that two regimes may operate. The first is where laccase is bonded to both the GCE substrate and the growing laccase coating itself by a weak adsorption process and decrease in the analytical signal is caused by the washing out of weakly bonded laccase during measurements. What is seen from Figure 3a is that after an initial period of signal decline the signal stabilizes. This indicates that the corona SPP process of laccase biosensor construction may comprise two parts, an adsorption process and a second mechanism being the creation of stable covalent bonds with the GCE surface and/or within the laccase coating itself. Adsorbed laccase is removed during the Rutin quantitation measurements while laccase molecules connected by covalent bonds remain on the GCE surface and are responsible for generation of a long-term analytical signal. Biosensor stability can undoubtedly be improved by the introduction of interlayers composed of, for example, carbon nanomaterials to act as covalent bonding sites in adhesion promotion, and possibly by the introduction of polymerizing gas such as ethylene into the helium gas flow feeding the corona jet, a topic for future work. The study of biosensor storage conditions showed that contact of the corona SPP deposited laccase bio-recognition layer with certain solutions can cause its dissolution and reduced analytical signals. However, as mentioned in Section 3.3, storage of GCE/laccase_30_ in an environment of 1 M acetate buffer has an advantage in the form of reduction in measurement of background current. This perhaps results from hydrogen bonding between –OH groups of Rutin and –NH_2_ groups of laccase. Decrease in measurement of background current is also caused by washing out of Rutin molecules from laccase modified GCE during storage in acetate buffer environment.

An important factor influencing the applicability of new biosensors is their response time which determines measurement time and the applicability of a biosensor in practical use. In this work, this factor was investigated at three different Rutin concentrations, as described in Section 3.3, and a response time of 120 s for the GCE/laccase_30_ sensor was measured independent of analyte concentration. Typically, three factors influence response time: (1) diffusion rate and solubility of the analyte, (2) kinetics of the enzymatic reaction, and (3) electrochemical reduction of the oxidized form of the laccase bio-recognition layer [29]. According to studies [31] response time is dependent on the molecular weight of the analyte so that an increase of analyte molecular weight from 109.11 g/mol (2-amino phenol) to 548.7 g/mol (ABTS) caused an extension of response time from 120 ± 20 s to 400 ± 20 s respectively. The molecular weight of Rutin is 610.53 g/mol indicating that, according to work [31], the response time of the GCE/laccase_30_ sensor to Rutin should be longer than 400 s. In fact, the GCE/laccase_30_ sensor response time is very significantly less indicating that the application in laccase biosensor construction of the corona SPP method can improve the utility of biosensors through reduction in their response time. This property of the GCE/laccase_30_ sensor may well result from the simple structure of the as-deposited bio-recognition layer without the additional chemicals needed by conventional sensor fabrication methods.

Applicability of the GCE/laccase_30_ sensor has also been tested by determination of Rutin in three different pharmaceutical formulations. Data shown in Table 1 indicated that the SPP deposited laccase biosensor enabled the determination of Rutin concentration and obtained results that were in good agreement with the labeled amount. The fabricated biosensors have demonstrated their capability to determine Rutin concentration independent of the sample matrix containing the Rutin.

## 5. Conclusions

This paper presents a new corona SPP method of biosensor construction. Sensor fabrication has been done using laccase as the biological precursor but the technique can be extended to other enzymes. Laccase-based biosensors are interesting because of their wide application in pharmaceutical, food, etc. industries in the determination of many chemical compounds as well as in environmental applications in pollution monitoring. Developing new methods of biosensor construction is a huge challenge due to the need to meet two key requirements:

1. Minimal damage to and denaturating of the enzyme; and
2. Enabling fabrication of biosensors with good analytical performance parameters.

Currently used methods of biosensor construction are long, expensive and need many organic chemicals. The innovative corona SPP method presented in this work is a one-step, environmentally friendly method allowing deposition of the bio-recognition layer without using any additional chemicals (apart from H_2_O and ethyl alcohol). Optimization of the corona SPP process deposition time confirmed the importance of this process control parameter in determining linearity, sensitivity and signal stability of laccase biosensors and showed that the best deposition time within the range investigated is 30 s (GCE/laccase_30_). This biosensor showed the best stability of analytical signal and wide linear range of analytical response. Application of the GCE/laccase_30_ sensor for determination of Rutin in real pharmaceutical samples showed that laccase bio-recognition layers deposited by a corona plasma jet are characterized by similar or better analytical performance parameters than biosensors fabricated using conventional methods.

The results presented in this work indicate that plasma-based functional bio-coating deposition methods such as corona SPP can be successfully applied in biotechnology for biosensor construction.

## Figures and Tables

**Table 1:** Results of the determination of Rutin (n = 3) in pharmaceutical samples.

Sample	Declared (mg/Tablet)	Found by GCE/laccase_30_ (mg/Tablet)	Recovery (%)	Found by UV-Vis (mg/Tablet)
Rutinoscorbin	25.0	26.1 ± 0.4	104.0	26.4 ± 0.9
Cerutin	25.0	24.3 ± 0.5	97.2	27.2 ± 0.8
Vanescin	60.0	58.1 ± 0.6	96.8	59.3 ± 1.2

**Table 2:** Comparison of different biosensors designed for Rutin determination.

Enzyme	Basis Electrode	Modified Electrode	Linear Range (μmol/dm^3^)	Sensitivity (µA·dm^3^/µmol)	Ref.
Laccase (*Aspergillus oryzae*)	CPE ^a^	BMI-Tf_2_N ^c^-laccase	4.8–46.2	0.772	[28]
		DMI-Tf_2_N ^d^-laccase	5.84–53,6	0.277	
		TDMI-Tf_2_N ^e^-laccase	5.84–53.6	0.312	
Laccase (*Aspergillus oryzae*)	PGE ^b^	Laccase encapsulation inside of chitosan microspheres	6.0–3.9	3.19	[30]
			5.82–13.10	7.71	
Laccase (*Cerrena unicolor*)	GCE	GCE/laccase_10_	0.3–0.5	39.25	This work
			0.7–1.2	10.32	
Laccase (*Cerrena unicolor*)	GCE	GCE/laccase_15_	0.2–0.8	18.18	
			0.9–1.2	7.42	
Laccase (*Cerrena unicolor*)	GCE	GCE/laccase_30_	0.1–0.7	5.85	
			0.7–1.3	2.47	

^a^ CPE—carbon paste electrode, ^b^ PGE—printed graphite electrode, ^c^ BMI-Tf2N-1-butyl-3-methylimidazolium bis (trifluoromethylsulfonyl) imide, ^d^ DMI-1-decyl-3-methylimidazolium, ^e^ TDMI-1-tetradecyl-3-methylimidazolium.
